# Distinct bacterial and fungal communities linked to functional potential in fermented fish and vegetables

**DOI:** 10.3389/fmicb.2026.1850075

**Published:** 2026-06-03

**Authors:** Namrata Jiya, Shankar Prasad Sha, Wormirin Khudai, Shretima Yadav, Rohit Sasane, Sumit Prasad Sah, Kriti Ghatani, Avinash Sharma

**Affiliations:** 1 Microbial Ecology Laboratory, BRIC-National Centre for Cell Science, Pune, India; 2 Food Microbiology Laboratory, Department of Botany, Kurseong College, Darjeeling, West Bengal, India; 3 Regional Centre for Biotechnology, NCR-Biotech Cluster, Faridabad, India; 4 Food Microbiology Laboratory, Department of Food Technology, University of North Bengal, Raja Rammohunpur, India

**Keywords:** fermented foods, functional potential, lactic acid bacteria, microbiome, mycobiome, targeted metagenomics

## Abstract

**Introduction:**

Traditional fermented foods constitute a vital component of ethnic community diets; consequently, characterizing their specific food microbiome is essential for elucidating their nutritional, functional and health related attributes.

**Methods:**

In this study, targeted metagenomics was employed to investigate the bacterial and fungal compositions of fermented fish and vegetables from North Bengal, India. The functional predictions of the fermented food microbiomes was performed using PICRUSt2.

**Results and discussion:**

High throughput sequencing of 16S rRNA and ITS genes revealed substantial differences in the diversity indices amongst the fermented fishes and vegetables. Fish samples were dominated by Pseudomonadota (23.05%), whereas vegetables were enriched in Bacillota (32.17%), with *Psychrobacter* and *Aliivibrio* prevalent in fishes and lactic acid bacteria including *Levilactobacillus, Paucilactobacillus* and *Pediococcus* dominant in vegetables. The fungal genera *Bisifusarium* belonging to Ascomycota and *Cystobasidium* affiliated to *Basidiomycota*, were abundant in the fermented fishes and vegetables, respectively. Functional predictions of bacterial and fungal communities revealed enhanced carbohydrate metabolism, biosynthesis pathways related to vitamins, short-chain fatty acids, organic acids, proteolytic enzymes and compounds contributing to organoleptic attributes in these fermented foods. The assessment of microbial communities associated with the traditionally fermented foods of North Bengal revealed the key microbial taxa involved in the fermentation process and their nutritional properties.

## 1 Introduction

Fermentation is one of the oldest, traditional processes known to humanity that enhances the shelf life, safety and sensory quality of food. It involves the breakdown of complex carbohydrates by microorganisms into simpler substances like alcohols and organic acids (Herlina and Setiarto, 2025). The International Scientific Association for Probiotics and Prebiotics (ISAPP) have defined fermented foods as the items prepared by desired microbial growth and enzymatic breakdown of its constituents (Marco et al., 2021). The major microorganisms driving the fermentation processes include the lactic acid bacteria like *Lactobacillus, Lactococcus, Lactiplantibacillus, Leuconostoc, Pediococcus* and beneficial fungi like *Aspergillus, Mucor, Penicillium* and *Rhizopus* (Tamang, 2022; Sha et al., 2018; Alam et al., 2024). These contribute aroma, flavor, nutritional quality and probiotic properties to the fermented foods. Probiotics are the microorganisms that when consumed in adequate amounts, confer health benefits like improved gastrointestinal health by reducing gut inflammation, enhanced immunity by production of vitamins and short-chain fatty acids (SCFAs), modulation of the gut microbiota by promoting beneficial microorganisms and by degrading antinutritional factors of foods (Jani and Sharma, 2021; Ghatani et al., 2024; Valentino et al., 2024). The Food Safety and Standards Authority of India (FSSAI), have listed the probiotic microorganisms that can be used in fermentation of commercial products (Food Safety Standards, 2022).

Fermented foods are consumed popularly worldwide. For instance, yogurt and kefir are associated with improved digestive health in Middle Eastern and Eastern European cultures, while *kimchi*, a Korean fermented dish, has been reported to reduce blood glucose levels (Kok and Hutkins, 2018). In India as well, the traditional fermented foods are prepared using the natural fermentation process so as to preserve the locally available, seasonal items like fish, vegetables and crops (Tamang et al., 2021; Sha et al., 2017). Indian fermented foods such as *Axone* and *Hawaijar* are soyabean plant-based fermentation products, well known for their high protein content and probiotic properties, are considered beneficial for cardiovascular health, whereas *Soibum*, a fermented bamboo shoot product, is consumed for its antioxidant and probiotic effects by the ethnic communities of North East India (Tamang et al., 2020; Jani and Sharma, 2021). Native populations of North Bengal region produce certain fermented products using fishes and vegetables by salt preservation. The utilization of salt for preservation selectively promotes the growth of halotolerant microorganisms while inhibiting the undesired microorganisms or pathogens (Belleggia and Osimani, 2023). Beyond their biological importance, these fermented foods are commercially traded in local markets, providing a critical socio-economic foundation for native population.

However, since traditional fermentation is carried out under non-sterile, home-based conditions, the risk of contamination and inconsistent microbial composition remains a concern, potentially compromising the safety and efficacy of the products (Das et al., 2024). It has also been proven that similar fermentation processes display distinct region-specific characteristics which cause variations in their constituent microbial communities (Herlina and Setiarto, 2025). The European Food Safety Authority (EFSA) guidelines have stated the key points while using microorganisms as starter cultures in food fermentation keeping in mind the safety concerns and risk of antimicrobial resistance in the microorganisms, which is an increasing global crisis faced by humanity (Sharma et al., 2023; Todorovic et al., 2024). Therefore, understanding the microbial communities involved in these traditional fermentation processes is crucial for enhancing food safety, ensuring consistent product quality and uncovering their potential health benefits. To gain deeper understanding of these fermentation ecosystems, we investigated the bacterial and fungal communities associated with fermented fish and vegetable foods of North Bengal using targeted high throughput sequencing. This investigation provides insights into the role of microbial communities driving fermentation processes, the taxonomic composition, and functional attributes.

## 2 Materials and methods

### 2.1 Sampling

**Figure 1:**
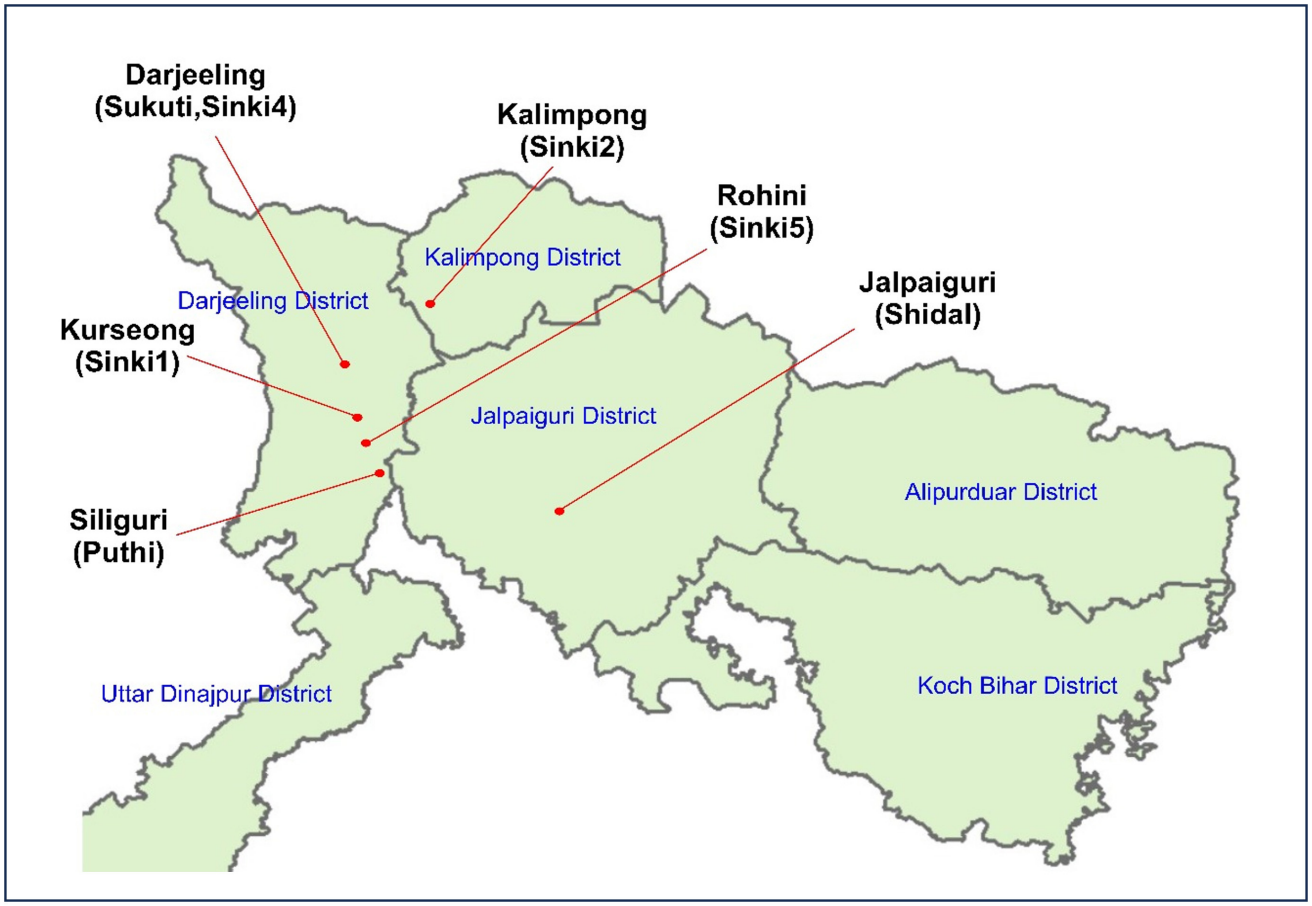
Region-specific fermented foods marked site-wise on the map of North Bengal, India.

The three fermented fishes included *Sukuti* (FF2), *Puthi* (FF3) and *Shidal* (FF4). *Sukuti mach* is a native fermented fish delicacy of Darjeeling hills, prepared traditionally by washing the fishes, mixing them with salt, turmeric and other spices followed by sun-drying them for 2 weeks. *Puthi* is a locally consumed, fermented fish of North Bengal. *Shidal* is a fermented fish cake prepared by washing, sun-drying and grinding the fish using mortar (traditionally called *Okhali*) and pestle. The freshly ground fish powder is then mixed with ginger, garlic, onion and several other spices and kept for fermentation for 4 to 6 h. This fermented mixture is then shaped into circular cakes and sun-dried for a couple of days. *Shidal* is mainly consumed by the Rajbangshi community of North Bengal. Each of the fermented fish had a characteristic unique strong smell or aroma. The remaining four samples under our study consisted of traditionally fermented leafy vegetable radish taproot (*Raphanus sativus* L.) samples, designated as *Sinki*. For its preparation, the radishes are allowed to wilt for 3 days, crushed coarsely and placed in 2–3 feet deep pits for pit fermentation to occur naturally for 22 to 30 days. The freshly fermented *Sinki* are then cut into small pieces and dried under the sun for 3–5 days. The process of *Sinki* fermentation occurs with variations in the different regions of North Bengal therefore we have collected the fermented vegetable *Sinki* samples from four towns named as Kurseong (Sin1), Kalimpong (Sin2), Darjeeling (Sin4) and Rohini (Sin5), located in North Bengal, India where the fermentation processes varied from each other. *Sinki* are widely consumed by the Nepali community in this region. The seven fermented food items were collected in triplicates from the local households/markets (*Haat*) located in various regions of North Bengal, India (Figure 1).

### 2.2 Targeted amplicon sequencing

The collected fermented food items were cut into small pieces and crushed using sterile pestles followed by extracting the community DNA using QIAamp DNA Micro kit (Qiagen, The Netherlands) as per the manufacturer's protocol. The quality and quantity were checked using the Nanodrop One spectrophotometer (Thermo Fisher Scientific, USA). DNA extraction was performed in triplicates for each sample, and the resulting extracts were pooled into a single composite sample in equimolar concentrations prior to the sequencing. Targeted amplification of the V4 region of 16S rRNA gene was carried out using the primers 515F (GTGYCAGCMGCCGCGGTAA) and 806R (GGACTACNVGGGTWTCTAAT), while the ITS1 region was amplified using ITS1F (CTTGGTCATTTAGAGGAAGTAA) and ITS2R (GCTGCGTTCTTCATCGATGC) primer pairs as described previously (Jani and Sharma, 2021). The library preparation was performed using Illumina 16S rRNA metagenomic protocol and quantification was achieved using Invitrogen Qubit 4.0 fluorometer (Thermo Fisher Scientific, USA). The equimolar libraries were pooled and the targeted amplicon sequencing was carried out using the in-house Illumina MiSeq platform using v2 chemistry (2^*^250 bp).

### 2.3 Bioinformatics analysis

The raw data obtained on sequencing was checked using the FASTQC pipeline. The good quality reads were analyzed using the Divisive Amplicon Denoising Algorithm 2 (DADA2) pipeline in the R environment (version 4.4.2) (Callahan et al., 2016). The amplicon sequence variants (ASVs) generated as DADA2 output, were assigned taxonomy using the SILVA (v.138.2) and UNITE databases (version 10) for bacterial and fungal classification, respectively (Abarenkov et al., 2024; Chuvochina et al., 2026). The interpretation of results was achieved using various R packages like ggplot2, microbiome, phyloseq, RColorBrewer, readr, tidyverse and vegan in RStudio. The venn diagrams were constructed using InteractiVenn tool (Heberle et al., 2015). The functional predictions for both, bacterial and fungal communities were carried out using Phylogenetic Investigation of Communities by Reconstruction of Unobserved States 2, version 2.6.3 (PICRUSt2) (Douglas et al., 2020).

## 3 Results

### 3.1 Bacterial and fungal community profiling of fermented foods

**Figure 2:**
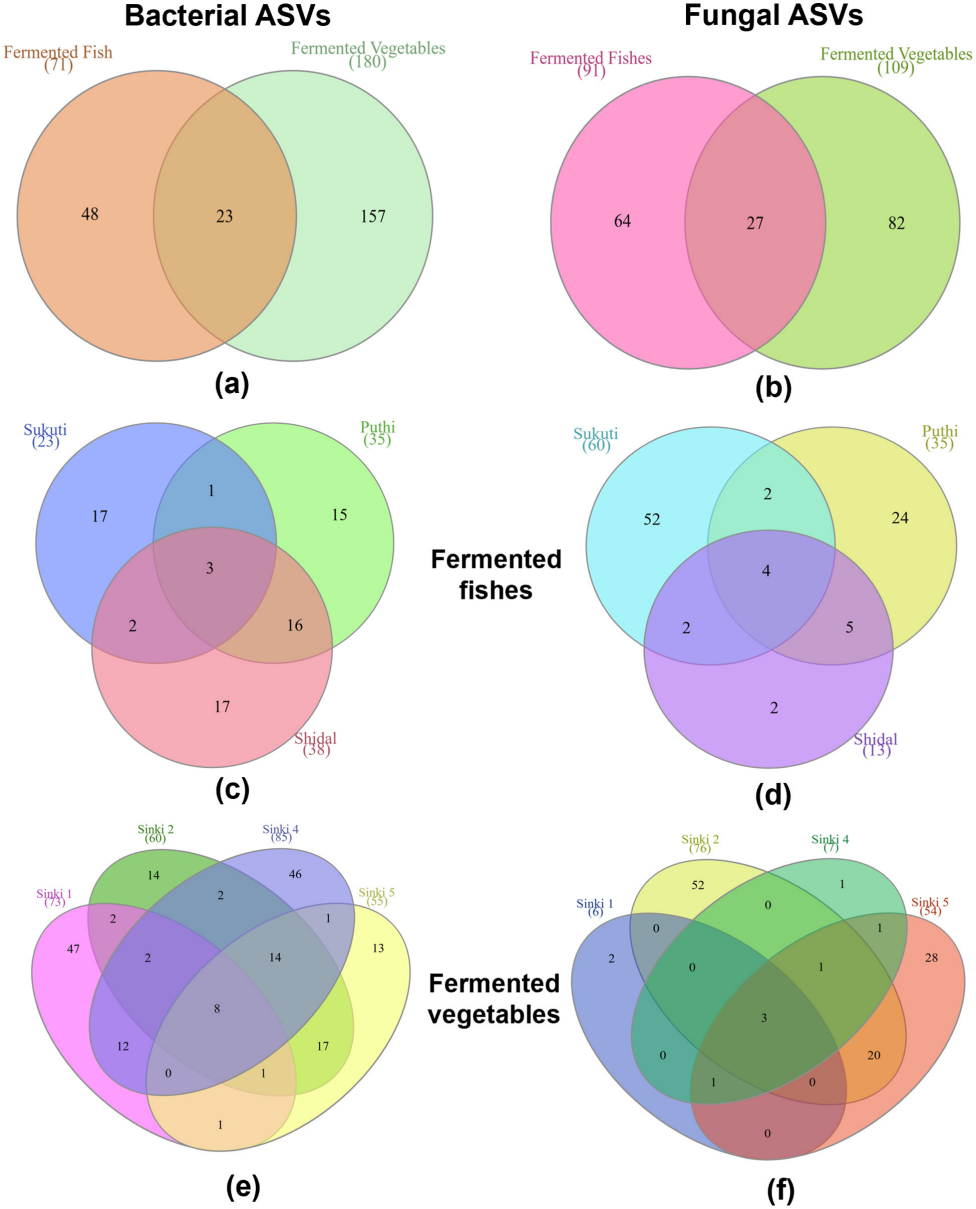
Venn diagram representing the unique and common ASVs at the genus level. **(a)** Between the fermented fishes and fermented vegetables; **(b)** Amongst the three fermented fishes of *Sukuti, Puthi* and *Shidal* and **(c)** Amongst the four vegetables, *Sinki*1, *Sinki*2, *Sinki*4 and *Sinki*5.

We compare the microbial diversities of fermented fishes and vegetables using 16S rRNA and ITS gene sequencing in our study. High throughput sequencing using the Illumina MiSeq platform resulted in 1019679 and 939782 raw reads which after filtering, trimming and merging with the DADA2 pipeline, resulted in 703086 and 517364 paired, non-chimeric reads for the bacterial and fungal sequencing, respectively. The unique ASVs in the fermented foods at the genus level, were determined using venn diagrams. The fermented fishes harbored 48 unique bacterial genera like *Psychrobacter, Aliivibrio, Pseudoalteromonas*, etc. and the fermented vegetables had 157 unique genera like *Weisella, Pediococcus, Levilactobacillus, Paucilactobacillus, Latilactobacillus, Companilactobacillus*, etc. Both, the fermented fishes and vegetables, shared 23 ASVs at the genus level which included *Vibrio, Staphylococcus, Clostridium* sensu stricto 1, *Klebsiella*, etc. The common and uniquely shared bacterial genera are represented in the Figure 2a. The 64 fungal genera, present uniquely in the fermented fishes included *Bisifusarium, Paraxerochrysium, Linnemannia, Neopestalotiopsis*, etc. while the fermented vegetables distinctively harbored 82 genera belonging to *Cystofilobasidium, Papiliotrema, Cutaneotrichosporon, Hannaella*, etc. The commonly detected fungal genera (*n* = 27) were *Aspergillus, Wallemia, Cladosporium, Alternaria, Sterigmatomyces, Nigrospora, Fusarium, Pichia, Rhodotorula*, etc. (Figure 2b). Similarly, the unique as well as overlapping bacterial and fungal genera between the two types of fermented foods (fishes and vegetables) were also determined (Figures 2c–f). Bacteria like *Lactobacillus, Lysinibacillus* and *Macrococcus* were present commonly in *Puthi, Shidal* and *Sukuti* fish samples. Similarly, the *Sinki* samples Sin1, Sin2, Sin4 and Sin5, retained *Bacillus, Lactiplantibacillus, Levilactobacillus* and *Pantoea* as the prevalent genera. Fungal genera commonly detected in the fishes belonged to *Aspergillus, Cladosporium, Debaryomyces* and *Penicillium* while in the fermented vegetables, *Aspergillus, Wallemia* and *Pichia* were identified.

**Figure 3:**
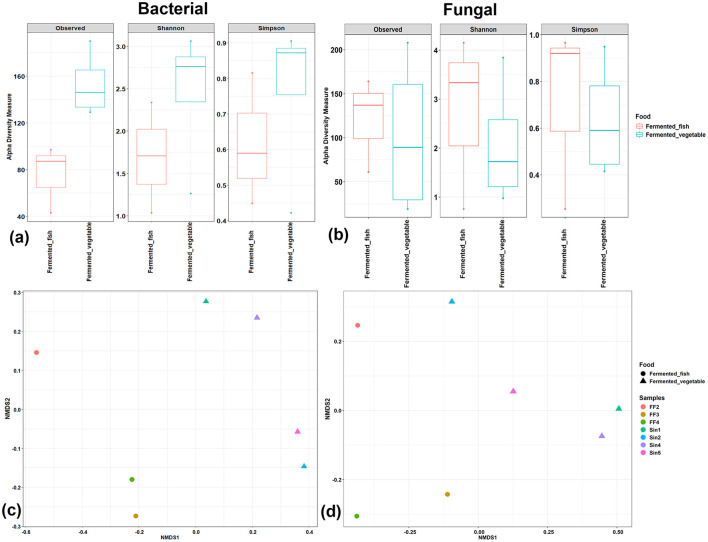
Alpha diversity indices indicating the variations between the fermented fishes and fermented vegetable samples, for the **(a)** bacterial and **(b)** fungal communities. NMDS plots showing the alterations in beta diversity of fermented fishes and vegetables with respect to **(c)** bacterial and **(d)** fungal diversities.

The two groups of fermented fishes and vegetables comprised of distinct bacterial and fungal communities as evident from the alpha and beta diversity indices. The Observed ASVs, Shannon and Simpson indices of the fermented fishes for the bacterial communities showed less diversity and richness for the three samples, whereas in the fermented vegetables or *Sinki* samples, higher diversity, richness and evenness were observed as depicted in the Figure 3a. However, in the case of fungal communities, a reverse trend was detected with higher observed ASVs, Shannon and Simpson diversity values in the fermented fishes than the fermented vegetables (Figure 3b). The beta diversity calculated using Bray- Curtis dissimilarities and depicted using non-metric multidimensional scaling (NMDS) showed distinct bacterial and fungal communities constituting the fermented fishes and vegetables. In the bacterial analysis, the fermented fishes FF3 (*Puthi*) and FF4 (*Shidal*) showed similar diversity as depicted by their clustering. Similarly, Sin2 and Sin5 as well as Sin1 and Sin4 had similar bacterial compositions (Figure 3c). In the case of fungal diversity, fermented fishes displayed scattering pattern which depicts distinction in their fungal communities, while in the fermented vegetables, Sin1 and Sin4 showed clustering indicative of similar fungal diversity (Figure 3d).

**Figure 4:**
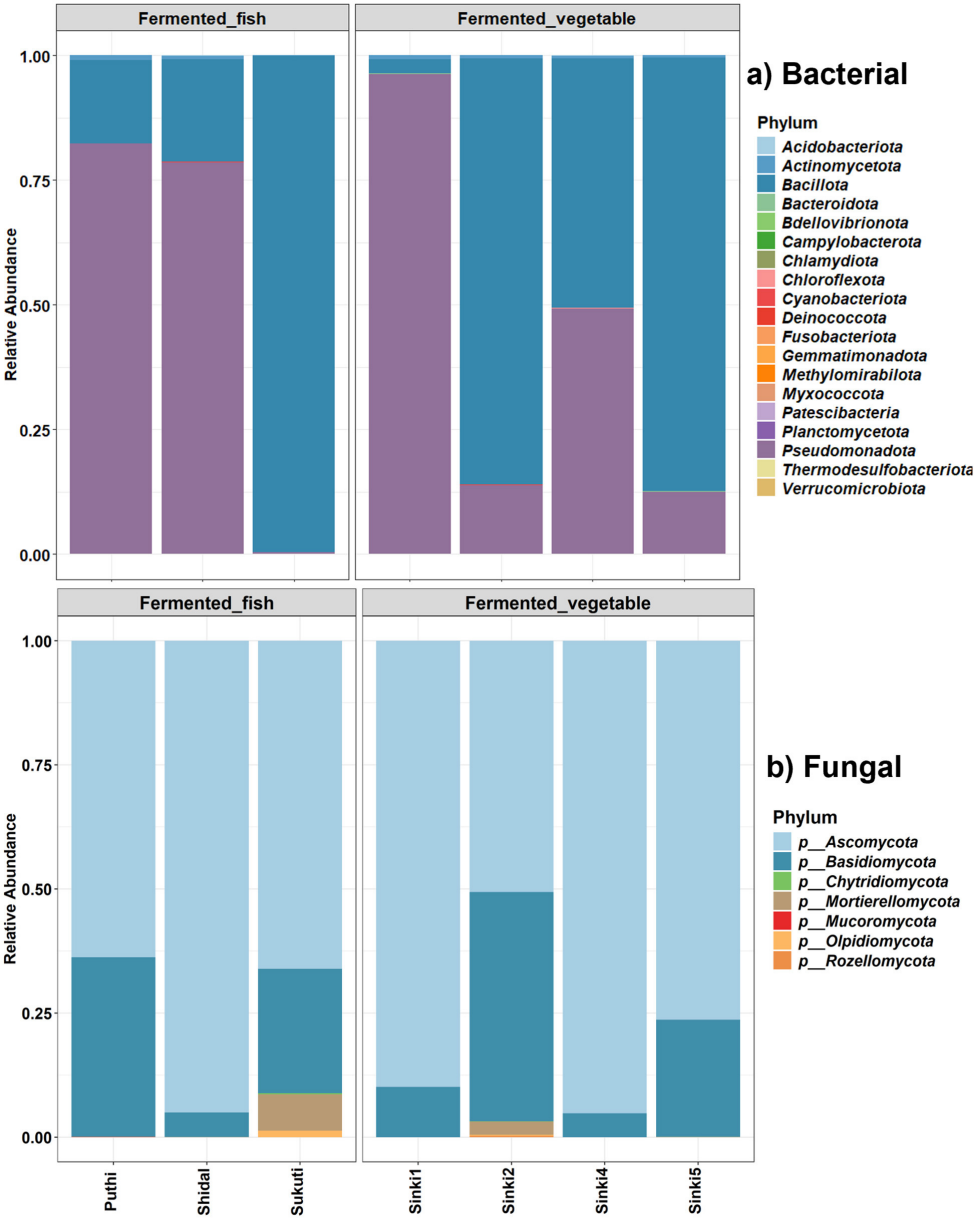
The microbial diversity at the phylum level depicting all the **(a)** bacterial and **(b)** fungal phyla present in the fermented foods.

The high throughput sequencing of fermented fish and vegetable samples revealed the presence of heterogenous bacterial and fungal communities responsible for the fermentation processes. A total of 19 bacterial phyla were detected in the fermented fishes (*Sukuti, Puthi* and *Shidal*) and vegetable (*Sinki*) samples. These included Pseudomonadota (23.05%), Bacillota (19.56%), and Actinomycetota (0.23%) present abundantly in the fermented fishes; while fermented vegetables were dominated by Bacillota (32.17%), Pseudomonadota (24.54%) and Actinomycetota (0.32%) (Figure 4a). The fungal community structure also showed distinctions at various taxonomic ranks. At the phylum level, Ascomycota (32.13%), Basidiomycota (9.44%) and Mortierellomycota (1.06%) were majorly found in the fermented fishes, and the fermented vegetables contained Ascomycota (44.57%) and Basidiomycota (12.1%) abundantly (Figure 4b).

**Figure 5:**
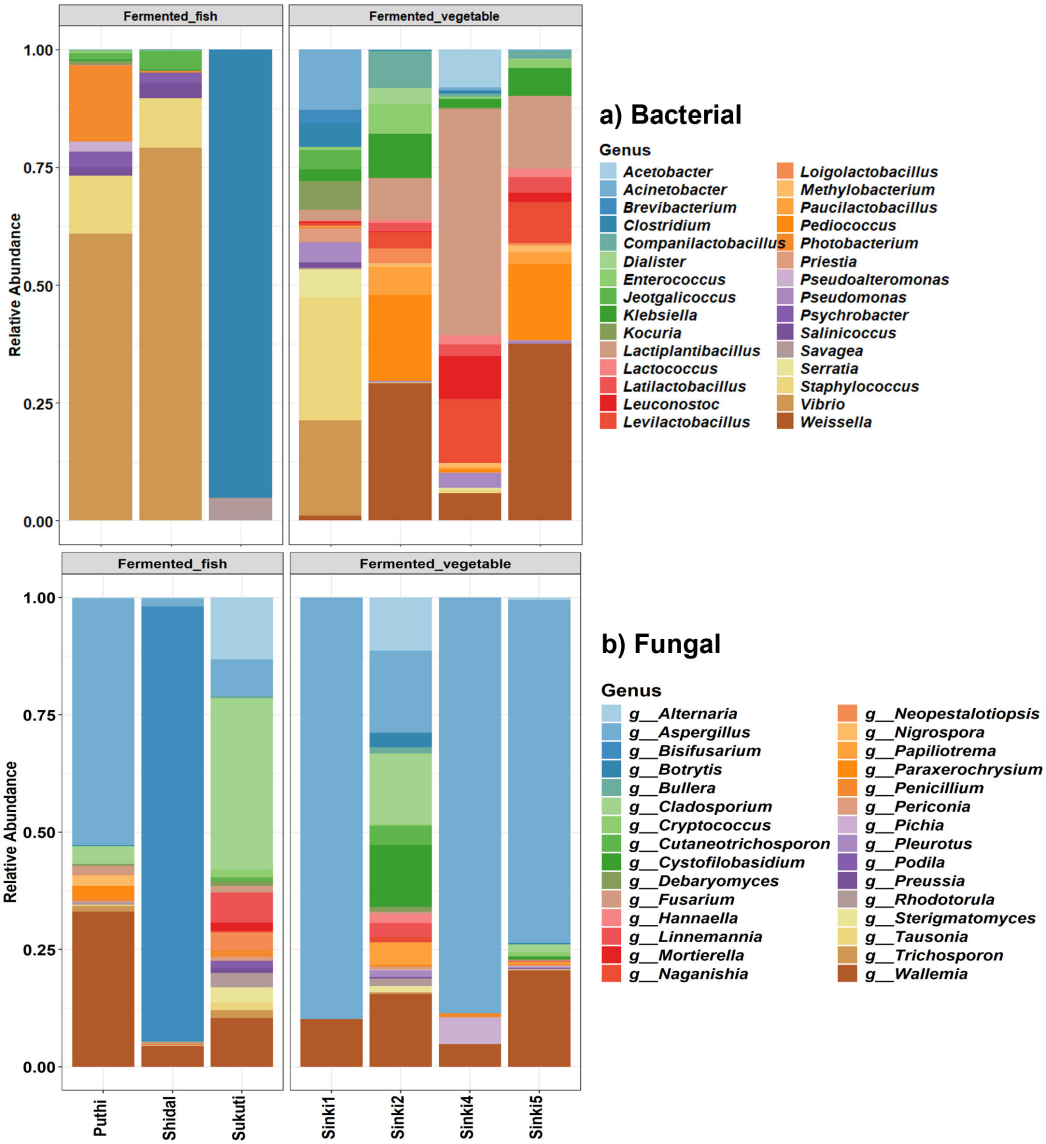
The microbial diversity at the genus level depicting the top 30 **(a)** bacterial and **(b)** fungal genera determined in the fermented food items.

At the genus level, the bacterial genera detected prevalently in the fermented fishes were *Vibrio* (18.98%), *Clostridium* (13.32%), *Staphylococcus* (3.1%) and *Photobacterium* (2.2%) (Figure 5a). The fermented vegetable products *Sinki* harbored *Lactiplantibacillus* (10.17%), *Weisella* (9.73%), *Pediococcus* (4.62%), *Levilactobacillus* (3.59%), *Staphylococcus* (2.95%), *Klebsiella* (2.52%) and *Vibrio* (2.2%) as the major bacterial genera, also supported by Tamang et al. (2016). The fungal genera prevalent in the fermented fishes were *Bisifusarium* (13.18%), *Aspergillus* (8.28%), *Wallemia* (6.34%), *Cladosporium* (4.86%) and *Alternaria* (1.63%). On the other hand, the fermented vegetables comprised of *Aspergillus* (37.85%), *Wallemia* (6.98%), *Cladosporium* (2.15%), *Cystofilobasidium* (1.73%) and *Alternaria* (1.51%) as the major fungal genera (Figure 5b).

### 3.2 Metagenomic imputation using PICRUSt2

**Figure 6:**
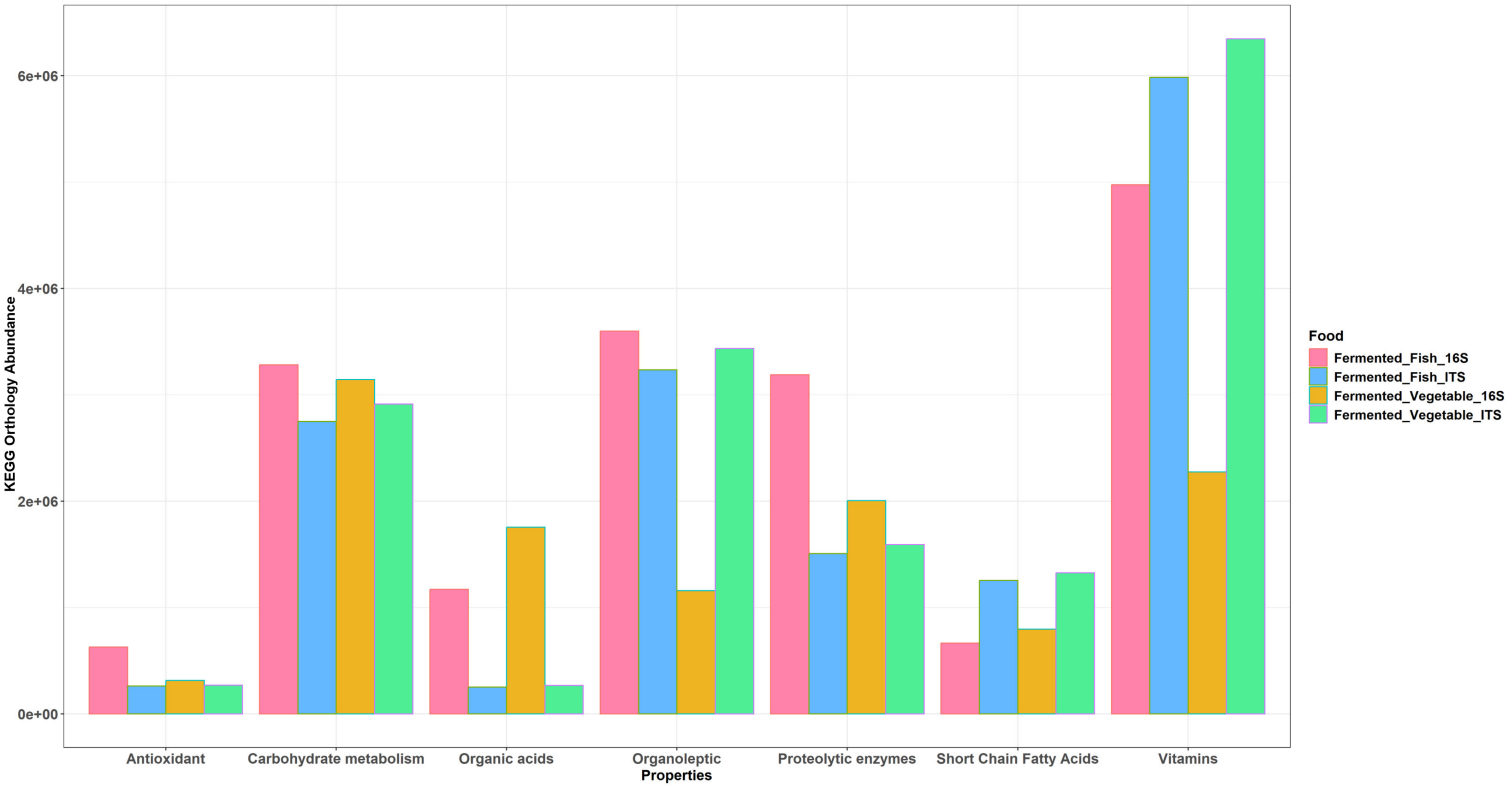
The major metabolic pathways detected for the bacterial (16S) and fungal (ITS) communities using PICRUSt2, depicting the properties associated with the fermented fishes and vegetables from our study.

Functional prediction based on the 16S rRNA and ITS gene sequences using PICRUSt2 revealed distinct metabolic profiles across the two types of traditionally fermented foods of North Bengal. The PICRUSt2 results uncovered the roles of bacteria and fungi, and the beneficial properties they confer to the fermented fishes and vegetables, like carbohydrate metabolism, production of antioxidants, organic acids, short chain fatty acids (SCFAs), vitamins and proteolytic enzymes as well as enhancement of organoleptic properties (Figure 6).

The fungal communities involved in the fermentation of vegetable and fishes, produced the highest amount of vitamins followed by the bacterial communities. The fishes had higher abundance of pathways for the organoleptic properties (like aromatic nature due to branched chain amino acids (BCAA), glutamate/aspartate, aldehydes, etc.) for both bacterial and fungal communities, while the vegetables had higher BCAA producing pathways contributing to organoleptic properties, in the case of fungal communities. Proteolytic enzymes like peptidases and proteases, which cause protein degradation, increase the bio-availability of amino acids and lead to flavor formation in the fermented foods, were produced plentifully by bacterial communities of fishes and vegetables. Increased production of SCFAs like acetate, propionate and butyrate, which improve the gut health and act as mediators of the gut-brain axis, was observed in the fungal and bacterial communities of both the food types (Silva et al., 2020; Liu et al., 2024). Organic acid production was observed abundantly in the bacterial communities associated with the fermented fishes and vegetables in our study. Furthermore, the bacterial population of fermented fishes showed higher levels of antioxidant enzymes such as superoxide dismutase and glutathione synthase, indicating a greater response to oxidative stress, than fermented vegetables. Supporting the functional predictions, carbohydrate metabolism which uncovered the enzymes responsible for glycolysis the first step of fermentation (glucokinase, phosphofructokinase, glyceraldehyde-3-phosphate dehydrogenase, etc.), degradation of complex carbohydrates (amylases, glucanases) and probiotic properties (beta-galactosidases) were highly abundant in both bacterial and fungal communities of fermented fish and vegetable samples (Dupont et al., 2017; Belleggia and Osimani, 2023). Thus, the detection of these important metabolic pathways and their metabolites like vitamins, SCFAs and organic acids during the fermentation of fishes and vegetables signify the roles played by the accompanying bacterial and fungal communities.

## 4 Discussion

The comparison of microbial diversities of fermented fishes and fermented vegetables using 16S rRNA and ITS gene sequencing revealed distinct bacterial and fungal signatures associated with the diverse fermented foods of North Bengal, India. The taxonomic assignment resulted in high abundances of phyla Pseudomonadota (previously Proteobacteria) and Bacillota (previously Firmicutes) in the fermented fishes and vegetable in our study. Similarly, these two phyla play significant roles in the traditional fermentation of Indian fish products like *Hentak, Ngari, Shidal, Sidra, Suka ko maacha, Sukuti, Tungtap*, etc. (Tamang et al., 2016; Bhutia et al., 2021). Culture dependent and independent approaches have revealed members of Bacillota (especially the LAB) and Pseudomonadota as the major phyla in fermented vegetable products of cabbage, carrot and turnip (Cantuti Gendre et al., 2025). The mycobiome in fermented fishes and vegetables were dominated by phyla Ascomycota, Basidiomycota and Mortierellomycota. Along with its ecological importance, the phylum Ascomycota contributes significantly to fermentation of alcoholic beverages, bread, cheese and fish products (Dupont et al., 2017; Zhag et al., 2018). Members of Basidiomycota have been deployed for novel fermentation approaches for co-cultivation of mushrooms which improves the degradation of harmful aflatoxins found in nuts, thereby improving the food quality (Berger and Ersoy, 2022). Mortierellomycota isolates have been found in the fermented food items like *Sauerkraut*, rice wine, etc. (Wang et al., 2023). Thus, the various roles played by these fungi in the fermentation processes reported previously, are in agreement with our results.

The fermented fishes harbored bacterial genera like *Vibrio, Clostridium, Psychrobacter, Aliivibrio* and *Pseudoaltermonas* which have been related with several fermented fish products across the world (Bhutia et al., 2021; Belleggia and Osimani, 2023). The bacteria like *Lactobacillus, Lysinibacillus* and *Macrococcus* found in the fermented fishes, also represent the bacteria associated with the fermentation of *Suanyu*, a Chinese fermented fish product (Sun et al., 2022). In India as well, *Clostridium* and *Vibrio* have been discovered in fermented fish products like *Ngari* and *Tungtap* (Belleggia and Osimani, 2023). *Vibrio* are common inhabitants of the aquatic ecosystems and are hence found in close conjunction with fish products. Prevalence of *Macrococcus, Lactococcus, Staphylococcus* and *Vibrio* in fermented fish products like *Sheedal, Suanyu* and *Telesech* correlates with our study which corroborates their role in fermentation process (Zhag et al., 2018; Majumdar and Gupta, 2020; Bhutia et al., 2021). *Photobacterium* spp. found majorly in the Puthi sample, have also been reported in Chinese and Korean fermented fish products (Belleggia and Osimani, 2023). *Lactococcus* spp. are known to produce nisin, an antimicrobial peptide having a broad-spectrum effect, which acts as a natural preservative in the fermented foods (Kitagawa et al., 2019).

The lactic acid bacteria (LAB) comprising *Weisella, Pediococcus, Levilactobacillus, Paucilactobacillus, Latilactobacillus, Companilactobacillus*, etc. found exclusively in the fermented vegetables in our study, have been linked to fermentation of vegetables like cabbage, beetroot, capsicum, etc. (Yuan et al., 2023). LAB have been used as starter cultures for initiating the fermentation of vegetables, which in turn enhance the flavor and texture of foods by the production of exopolysaccharides and beneficial metabolites (Jurášková et al., 2022). These LAB convert complex carbohydrates to lactic acid that increases acidity and drives the fermentation process (Ali et al., 2025).

In our study fungal communities also played important metabolic roles in the fermentation of fishes and vegetables. Members of the genus *Bisifusarium*, found most abundantly in the fermented fishes, have been isolated from cheese fermentation processes previously (Savary et al., 2023). The fungi like *Cystofilobasidium, Papiliotrema, Cutaneotrichosporon, Hannaella*, etc., present in the fermented vegetables were identified to play important roles at various stages of wine fermentation (Abdo et al., 2020). *Aspergillus* comprises the fungi driving fermentation of several Asian fermented products like *Meju, Ngari, Gajami- Sikhae*, etc. by producing proteolytic enzymes which breakdown fish proteins, enhance nutritional content and lead to aroma as well as flavor development (Tamang, 2022; Belleggia and Osimani, 2023). Similarly, *Wallemia* have been found to carry out the fermentation of soybean products like *Doushen* and *Kinema* (Luo et al., 2020).

Although fermented foods are widely recognized for their health benefits, their production remains largely artisanal and empirical, often lacking standardization and process control. Differences in raw substrates, processing conditions, storage, packaging, and sanitation can result in inconsistent product quality. Consequently, contamination by spoilage organisms or pathogenic microorganisms may occur through raw materials, processing equipment, environmental exposure, fermentation, transportation, or distribution (Skowron et al., 2022; Ramos et al., 2023; Owolabi et al., 2022). Pathogenic genera, including *Bacillus, Clostridium, Staphylococcus*, and *Vibrio*, have been detected in traditionally fermented foods (Skowron et al., 2022; Oyedeji et al., 2023). Similarly, *Vibrio, Klebsiella, Photobacterium*, and *Acinetobacter* have been reported in street vended foods across different regions, highlighting their contribution to foodborne contamination (Cárdenas et al., 2024). Consistent with these reports, the present study detected potentially pathogenic bacterial genera in fermented fish, including *Vibrio* (18.9%), *Clostridium* sensu stricto 1 (13.3%), *Staphylococcus* (3.1%), and *Photobacterium* (2.2%). Fermented vegetables contained *Staphylococcus* (2.9%), *Klebsiella* (2.5%), *Vibrio* (2.2%), and *Acinetobacter* (1.5%) as potentially pathogenic genera. Fermented fish exhibited a greater abundance of potentially pathogenic taxa than fermented vegetables.

Consumption of food contaminated with potentially pathogenic *Vibrio* spp. can lead to gastrointestinal infections characterized by vomiting and diarrhea. *Vibrio* spp. are also detected in raw meat, food dressings, and seafood, and are known to cause vibriosis, particularly following the consumption of raw or undercooked seafood (Cárdenas et al., 2024). In addition, *Vibrio cholerae* is a well-established causative agent of foodborne diarrheal disease (Havelaar et al., 2015). *Vibrio* and *Photobacterium* are recognized contributors to fish spoilage; however, *Photobacterium damselae* has also been implicated in skin infections and histamine fish poisoning in humans (Belleggia and Osimani, 2023; Cárdenas et al., 2024). *Clostridium* species have been reported in fermented fish products from India, Egypt, Canada, and Thailand and are linked to illnesses ranging from gastrointestinal disturbances to severe clinical disease (Belleggia and Osimani, 2023; Rodpai et al., 2021). *Clostridium botulinum* and *Clostridium perfringens* are causative agents of botulism and food poisoning, respectively (Farrukh et al., 2025). *Acinetobacter* species are of public health concern because of multidrug resistance and their association with gastroenteritis, bacteremia, peritonitis, endocarditis, and diarrhea (Cárdenas et al., 2024).

*Klebsiella pneumoniae* is recognized as a potentially virulent and antibiotic-resistant bacterium capable of serving as a reservoir of antimicrobial resistance genes within the food chain. It has been linked to gastrointestinal colonization and liver abscesses, particularly in Asian populations, and may facilitate horizontal transfer of resistance determinants to other microorganisms (Hartantyo et al., 2020). In addition, high alcohol-producing strains of *K. pneumoniae* have been implicated in non-alcoholic fatty liver disease (Xue et al., 2023). Contamination of fermented foods with *Staphylococcus* species, including *Staphylococcus aureus*, has been reported in fermented fish and meat products from Thailand and in *Shidol*, a traditional fermented product from Northeast India (Pop et al., 2024). *Staphylococcus aureus* is a multidrug-resistant foodborne pathogen associated with bacteremia and infective endocarditis (Farrukh et al., 2025). The halotolerant nature of Staphylococci supports their persistence in high-salt fermented fish products (Belleggia and Osimani, 2023). Coagulase-negative Staphylococci, including *Staphylococcus epidermidis*, are frequently detected in animal-derived foods and may harbor antibiotic resistance genes, representing an additional public health concern (Wolfe, 2023). Staphylococcal food poisoning is characterized by abdominal pain, vomiting, diarrhea, and fever (Pop et al., 2024). Although targeted amplicon sequencing reveals the presence of these potentially pathogenic taxa, its taxonomic resolution does not permit definitive strain-level identification. These findings provide a baseline for further investigation through deeper sequencing and targeted validation assays to determine the pathogenic potential and safety profiles of the detected taxa.

The prediction of functional potentials of bacterial and fungal communities of the fermented foods using PICRUSt2 led to detection of several beneficial pathways involved in carbohydrate metabolism, vitamins and short chain fatty acids production, and development of aromatic and organoleptic properties. The vitamins synthesized by the bacterial and fungal communities comprised of the B complex vitamins like cobalamin (vitamin B12), riboflavin (vitamin B2), pyridoxine (vitamin B6) which were produced in higher amounts than biotin (vitamin B7), folate (vitamin B9) and nicotinamide (vitamin B3). Bacterial genera including lactic acid bacteria in vegetables and *Clostridium* in fishes found in our study, produce essential B-complex vitamins (Keyvan et al., 2025). The presence of fungi like *Aspergillus* and *Pichia* in *Sinki* vegetable has been linked to increased production of riboflavin (Tambe and Chandekar, 2011). Nath et al. (2024) identified vitamins like riboflavin, α-tocopherol and thiamine, in *Napham* a traditional fermented fish paste from Assam, India; which is in alignment with our study where cobalamin (vitamin B12) emerged as the most abundant vitamin, followed by folate (vitamin B9), riboflavin (vitamin B2) and biotin (vitamin B7). Microorganisms like *Psychrobacter, Staphylococcus* and *Aspergillus* dominantly found the fishes, showed enhanced lipolytic and proteolytic activities leading to amino acid metabolism and flavor development during the fermentation processes (Zhag et al., 2018; Bhutia et al., 2021). Enzymes involved in amino acid metabolism- including aminopeptidases (encoded by genes like *pepA, pepP, pepT, pepN, pepQ, pepD, pepX, pepB, pepE*), glutamate decarboxylase, threonine dehydratase, aldehyde dehydrogenase, glutamate synthase, aspartate aminotransferase, 3-isopropylmalate dehydrogenase, ketol-acid reductoisomerase, leucine dehydrogenase and 2-isopropylmalate synthase were identified in both food types, with a notably higher abundance in the bacterial communities of fermented fishes, which was also observed by Belleggia and Osimani (2023). Likewise, the important microbial enzymes taking part in SCFA metabolism like acetate CoA-transferase, acetate kinase, butyrate kinase, propionate CoA-transferase, propionate kinase and lactate dehydrogenase were identified in both the fermented foods abundantly. The role of lactic acid bacteria like *Lactiplantibacillus, Lactobacillus, Levilactobacillus, Leuconostoc, Pediococcus* and *Weisella* has been noted in SCFA and organic acid production (Ali et al., 2025). Heterofermentative LAB associated with the fermented vegetables are central to organic acid production as they utilize key metabolic intermediates such as acetyl-CoA and pyruvate (Wu et al., 2021). Role of *Aspergillus* in organic acid production is very well established which also contributes to the sour taste of fermented foods (Wang et al., 2021; Wu et al., 2021; Yuan et al., 2023). Thus, utilizing a combined approach of taxonomic classification and functional predictions helped us elucidate the remarkable roles played by the bacterial and fungal communities associated with traditionally fermented foods of North Bengal, India.

In conclusion, this study characterizes the bacterial and fungal communities associated with traditionally important fermented foods such as *Sukuti, Puthi, Shidal* and *Sinki* from North Bengal. The findings provide valuable insights into key microbial taxa driving fermentation and their associated functional and health-related attributes. The predicted metabolic capabilities underscore the capacity of these microbial communities to produce beneficial metabolites, reinforcing the nutritional and functional relevance of traditional fermented foods. Collectively, this work provides a foundation for optimizing and preserving traditional fermentation practices and their associated microbiomes in ethnic food systems.

## Data Availability

The datasets presented in this study can be found in online repositories. The names of the repository/repositories and accession number(s) can be found below: https://www.ncbi.nlm.nih.gov/, PRJNA1256673 (SAMN48189686 to SAMN48189692 for 16S rRNA and SAMN48199445 to SAMN48199451 for ITS data).
